# MMT of HIV positive patients in Georgia

**DOI:** 10.1186/1742-4690-9-S1-P76

**Published:** 2012-05-25

**Authors:** Khatuna Todadze, Eka Kavtiashvili

**Affiliations:** 1 Research Institute On Addiction, Tbilisi, Georgia

## Introduction

The main route of HIV transmission is injective drug use in Georgia (58-60%). The most popular injective narcotics are opioids. Although prevalence of HIV among drug users is only 1-3%, the high number of IDUs and high prevalence of hepatitis C (from 65 to 80% according to the different studies) in this population could be the predictor of HIV increase. Methadone maintenance treatment (MMT) has been implementing throughout the country since 2005 as one of the important strategies to decrease drug related risky behavior, increase adherence to ARV treatment among HIV+ persons and improve the physical and psycho-social status of the patients.

## Materials and method

42 randomly selected HIV positive drug users undergoing MMT with intensive psychological counseling have been studied for 3 years. They received ARV therapy before inclusion in MMT at least 6 month. Risky behavior, quality of life, level of depression, anxiety and other data were measured before starting MMT and after 3, 6, 12, 18 months. The illegal use of psychotropic-narcotics was checked through random urine-testing 3 times per patient per month.

## Results

The study showed significant improvement of patients’ status. The remarkable decrease of depression and anxiety was observed (dynamic of average scores of depression - 24, 14, 14, 13, 14 and anxiety-46, 40, 40, 41, 39). Life quality increased in comparison with the starting data (76, 85, 86, 88, 93). The positive answers on psychotropic-narcotics were observed in 6.7% on average and even those patients didn’t admit any kind of injection-related risky behaviors.

## Conclusions

The analyses of data showed that combination of MMT, ARV and psychological counseling significantly improves the physical and psycho-social status of HIV positive IDUs, improves life quality and treatment adherence , dramatically decreases use of illegal psychotropic-narcotic drugs and decreases the risk of spread of HIV and other blood-transmitted diseases among injecting population in Georgia.

